# The approaching pilot for One Health governance index

**DOI:** 10.1186/s40249-023-01067-2

**Published:** 2023-03-13

**Authors:** Odel Y. Li, XiangCheng Wang, Kelly Yang, DongMei Liu, HuaChen Shi

**Affiliations:** 1 National Institute of Parasitic Diseases, Chinese Center for Disease Control and Prevention, NHC Key Laboratory of Parasite and Vector Biology, Shanghai, People’s Republic of China; 2 Shanghai Legislative Research Institute, Shanghai, People’s Republic of China; 3 School of Global Health, Shanghai Jiao Tong University School of Medicine, Shanghai, People’s Republic of China; 4 Queens College, The City University of New York, New York, NY USA; 5 Law School, Shanghai Jiao Tong University, Shanghai, People’s Republic of China; 6 Institute of Law, Shanghai Academy of Social Science, Shanghai, People’s Republic of China

**Keywords:** One Health, Governance, Developed countries, Developing countries, Rule of law

## Abstract

**Background:**

One Health approach advocates realizing the best health and harmonious symbiosis of human, animal and natural environment through cross-border, multi-sectoral and interdisciplinary cooperation. The good governance model is the leading factor for the performance of One Health governance. In order to tackle the complex problems in the One Health governance at the global level, the variation of One Health governance in different countries was analyzed by a set of indicators within the One Health system.

**Method:**

The capacity of One Health governance was assessed after establishment of a set of indicators for the One Health governance index (OHGI) following the methodology of the global One Health index. The data to calculate OHGI was collected from various database sources, including the Food and Agriculture Organization, the World Health Organization, the World Organization for Animal Health, and official health-related institutions of various countries. Eight indicators (including 19 sub-indicators) were employed in the OHGI system to comprehensively evaluate the capacity of One Health governance in 146 countries of the world.

**Results:**

Among the 146 countries scored in the OHGI system, the average score was 34.11, with a median score of 31.49, ranged from 8.50 to 70.28. Most countries with higher OHGI scores come from Europe and Central Asia, East Asia and the Pacific and North America, while countries with the lower OHGI scores are almost from sub-Saharan Africa. Six countries scored more than 65 points, including Australia, Sweden, Germany, Netherlands, the United States of America and Finland, indicating that these countries are relatively mature in most aspects of One Health governance. However, there were some developing countries with OHGI scored lower than 15. Therefore, the gap between countries with higher OHGI scores and those with lower OHGI scores is more than 60.

**Conclusions:**

Good governance on One Health is an important indicator to measure One Health’s governance capacity. The political stability, the level of rule of law and economic conditions in different regions are significantly correlated with the One Health governance capacity. Actions need to be taken urgently to close the gap of One Health governance between different regions.

**Supplementary Information:**

The online version contains supplementary material available at 10.1186/s40249-023-01067-2.

## Background

In 2021, the "One Health" senior expert group of World Organization for Animal Health, World Health Organization (WHO) and United Nations Environment Programme officially defined "One Health" as an integrated and unified approach aimed at sustainable balancing and optimizing the health of human, animals and ecosystems" [1]. One Health, as an approach to tackle the complex problems related to the health of humans, animals and our shared environment, has been paid much more attention than ever before, particularly after the COVID-19 pandemic [2]. One Health advocates to realize the optimal health outcomes in harmony with human, animal and natural environment through cross-border, multi-sectoral and interdisciplinary cooperation [3].

These inter-relationships of zoonotic diseases, antimicrobial resistance, foodborne illnesses, food insecurity, and climate change are essential to develop the health-related public policies [4]. Comprehensively social determinants (such as politics, economics, culture, social environment and other aspects) affect health in various fields. The good governance of One Health model has been proposed [5, 6]. However, there are gaps between public health governance in different countries [7].

Several articles have also called for action to promote the bio-social-ecological model to improve the health governance capacity [8–10], by which it is not only committed to achieving the optimal health outcomes, but also committed to eliminating health gaps and health inequality as well as integrating health in all policies [11]. Good governance for One Health must be realized jointly with the cooperation of multi-sectors, which need guidance by the laws, policies and multi-sectoral coordination [12]. At the global level, WHO, FAO and OIE have worked together to reach this goal ten years ago, despite less actions executed in the real world so far [13]. While at the country level, the major gap has been recognized as a dearth of the One Health governance framework from multi-sectoral cooperation based on local settings [14, 15]. One of the reasons for the existing gap is the lack of an efficient tool to evaluate the capacity of One Health governance [16, 17].

A framework for assessing health system governance (HSG) at national and sub-national levels is drawn on the experience of Four existing frameworks: WHO’s domains of stewardship; Pan American Health Organization’s (PAHO) essential public health functions; World Bank’s six basic aspects of governance; and United Nations Development Programme (UNDP) principles of good governance. The proposed HSG assessment framework includes the following 10 principles—strategic vision, participation and consensus orientation, rule of law, transparency, responsiveness, equity and inclusiveness, effectiveness and efficiency, accountability, intelligence and information, and ethics [18]. After several years of global health governance reform, most of the principles are still important for us to consider as the gateway to good governance. Therefore, we introduced some of these traditional evaluation indicators into One Health governance evaluation system.

Therefore, the main objective of the study was to establish an assessment framework for One Health governance as a part the global One Health index (GOHI, a recently established evaluation system for One Health performance) [16, 17]. To find out the gaps in One Health governance, it is possible to measure the capacity of good governance for One Health practice to find out the common difficulties of governance in various countries and to promote One Health practice at both global and national levels. This study also analyzed some characteristics of governance of BRICS countries and the top three countries.

## Method

### Research design

**Fig. 1:**
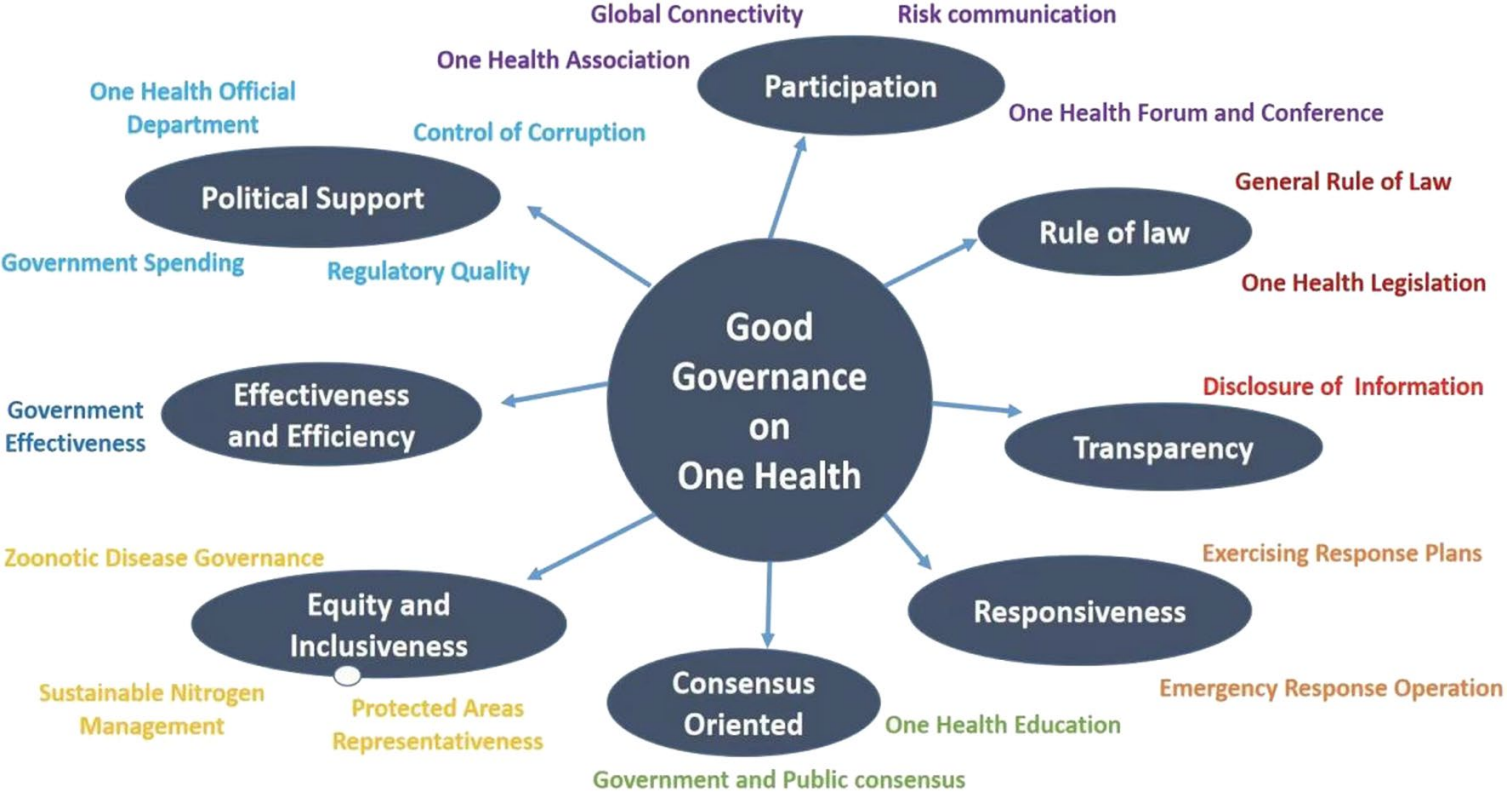
The OHGI indicators system (8 indicators and 19 sub-indicators). OHGI: One Health governance index

A four-pronged approach to the evaluation of One Health governance was performed based on the newly established GOHI for each country of the world [16, 17], namely One Health governance index (OHGI) system, including (i) The establishment of an OHGI database by extracting data from various database resources; (ii) The construction of an OHGI framework with three levels covering various indicators with their weights at each level based on a fuzzy analytical hierarchy process (FAHP) to assign the weights for most of the indicators (Fig. 1); (iii) The calculation of the OHGI score of each country in the world employing the established database of OHGI, following the method of the GOHI score calculation [17]; (iv) The comprehensive evaluation on the degree of global good governance for One Health in various countries measured by OHGI (Table 1).

**Table 1:** Stepwise for establishment of a OHGI indicator system

Input	Method	Output	Outcome
Indicators and levels	Expert advisory committee assumption according to the principles of relevance	Pathway	OHGI indicator system
Databases from various sources	Authoritative sources of official websites at the national level	Data	OHGI primary database
Self-evaluated data for some indicators	Searching the official data of 146 countries, including government official websites, national health departments, etc	Data	OHGI primary database
Calculation of weight for each indicator	A fuzzy analytical hierarchy process	Weight of each indicator at each level	OHGI score database
Score for each indicator at national level	GOHI score calculation	Total OHGI score for each country	Global ranking by OHGI score

*OHGI* One Health governance index, *GOHI* Global One Health index

### Database and resources

In order to set up the OHGI indicator system, a comprehensive database was established by searching literature, databases, group discussion, interviewing and consultation with experts. In the literature searching, a group of key words were selected, including “One Health”, “governance”, “legislation”, “legal strategy”, “health data management”, “public participation”, “transparency” and “effectiveness”. After several internal and international group discussions carried out for the best selection of the databases and literature, a large number of relevant databases have been searched, including databases from international resources or agencies, such as WHO, FAO, OIE, UN Sustainable Development Goals (SDG), World Bank, etc., as well as national official websites (Additional file 1). Some consultancy meetings were virtually arranged to interview with experts from international and national institutions to form a better framework of OHGI in accordance with the systemic structure-process-outcome (SPO) model for assessing management quality on One Health [19, 20].

To establish an OHGI database, two kinds of data were explored either by extracting from available databases or by innovative self-evaluated data from group discussions, consultancy meetings and web-searching in English. Those self-evaluated data focus on One Health related data, such as One Health forum, existing special legislation on One Health, existing One Health education system and One Health official institution. For self-evaluated data, a score of 0 or 1 has been given to each indicator (Additional file 2).

### Data analysis and pathway analysis

Each indicator was given a value by calculating data extracted from the established OHGI database. A logistical pathway of the indicator system was analyzed to improve the weighting approach for each indicator in the calculation of the total value of OHGI for each country (Additional file 2).

Based on the established database and framework of OHGI, a fuzzy analytical hierarchy process (FAHP) was used to assign the weights for the indicators and equal weight has been assigned to each sub-indicator under each indicator following the methods of the GOHI system [17]. The scores for each level of indicators were calculated and the country-based OHGI was ranked for all countries based on the total score of each country.

In addition, two case studies were performed to understand OHGI distribution among the top three countries and BRICs countries (Brazil, Russia, India, China and South Africa). The comparison of OHGI among those countries was analyzed to assess the variation of OHGI in different countries.

## Results

### OHGI framework

The OHGI framework is composed of eight dimensions, including public participation, rule of law, transparency, responsiveness, consensus orientation, fairness and inclusiveness, effectiveness and efficiency, and policy support. All those dimensions and relevant elements provided the bases of the OHGI framework (Fig. 1).

### OHGI indicator system

Under the OHGI framework, an indicator system of OHGI was created, covering a total of 27 indicators at two levels including 8 indicators and 19 sub-indicators (Table 2). Based on the data sources of each indicator, 14 sub-indicators extracted from available databases of the third partners, such as Huawei global connectivity index (GCI), Johns Hopkins University global health security index (GHS Index), Yale University environmental performance index (EPI), SDG report, The World Bank official data, etc. Meanwhile, 5 sub-indicators (e.g. One Health organization, One Health forum, specific legislation on One Health, One Health education and One Health government departments) was obtained by our research members who screening the official websites from 146 countries. Those official websites screened were from various institutions, e.g. national official education departments, national-level health departments, national universities, and international and non-government organizations. The details of the resources of sub-indicators were described in Additional file 2.

**Table 2:** The OHGI indicator system at two levels with their weights and resources

Indicator	Weight of indicator (%)	Sub-indicator	Weight of sub-indicator (%)
Participation	10.97	Global connectivity	25.00
		Risk communication	25.00
		One Health association	25.00
		One Health forums	25.00
Rule of law	15.75	General rule of law	50.00
		One Health specialized law and regulation	50.00
Transparency	9.98	Transparency	100.00
Responsiveness	12.56	Emergency response operation	50.00
		Exercising response plans	50.00
Consensus oriented	10.84	General Consensus oriented	50.00
		One Health education	50.00
Equity and inclusiveness	13.79	Zoonotic disease governance	33.33
		Protected areas representativeness	33.33
		Sustainable nitrogen management	33.33
Effectiveness and efficiency	13.18	Government effectiveness	100.00
Political support	12.93	One Health official department	25.00
		Control of corruption	25.00
		Regulatory quality	25.00
		Government spending	25.00

*OHGI* One Health governance index

Weighting for each indicator and sub-indicated has been performed which composed the indicator systems with a weighting scheme of OHGI framework (Table 2). Results showed that the weight of rule of law, as one of 8 indicators, was 15.75% out of 100% as the highest, followed by equity and inclusiveness (13.79%), effectiveness and efficiency (13.79%), political support (12.93%), responsiveness (12.56%), participation (10.97%), consensus oriented (10.84%) and transparency (9.98%). While, each of the sub-indicators has been assigned an equal weight under its indicator. For instance, weight of 25% was assigned for 4 sub-indicators under indicator the of participation, 50% weight for 2 sub-indicators under rule of law, 100% weight for 1 sub-indicators under transparency, 50% weight for 2 sub-indicators under responsiveness, 50% weight for 2 sub-indicators under consensus oriented, 33.33% weight for 3 sub-indicators under equity and inclusiveness, 100% weight for 1 sub-indicators under effectiveness and efficiency, 25% weight for 4 sub-indicators under political support.

### OHGI scores of each indicator

**Fig. 2:**
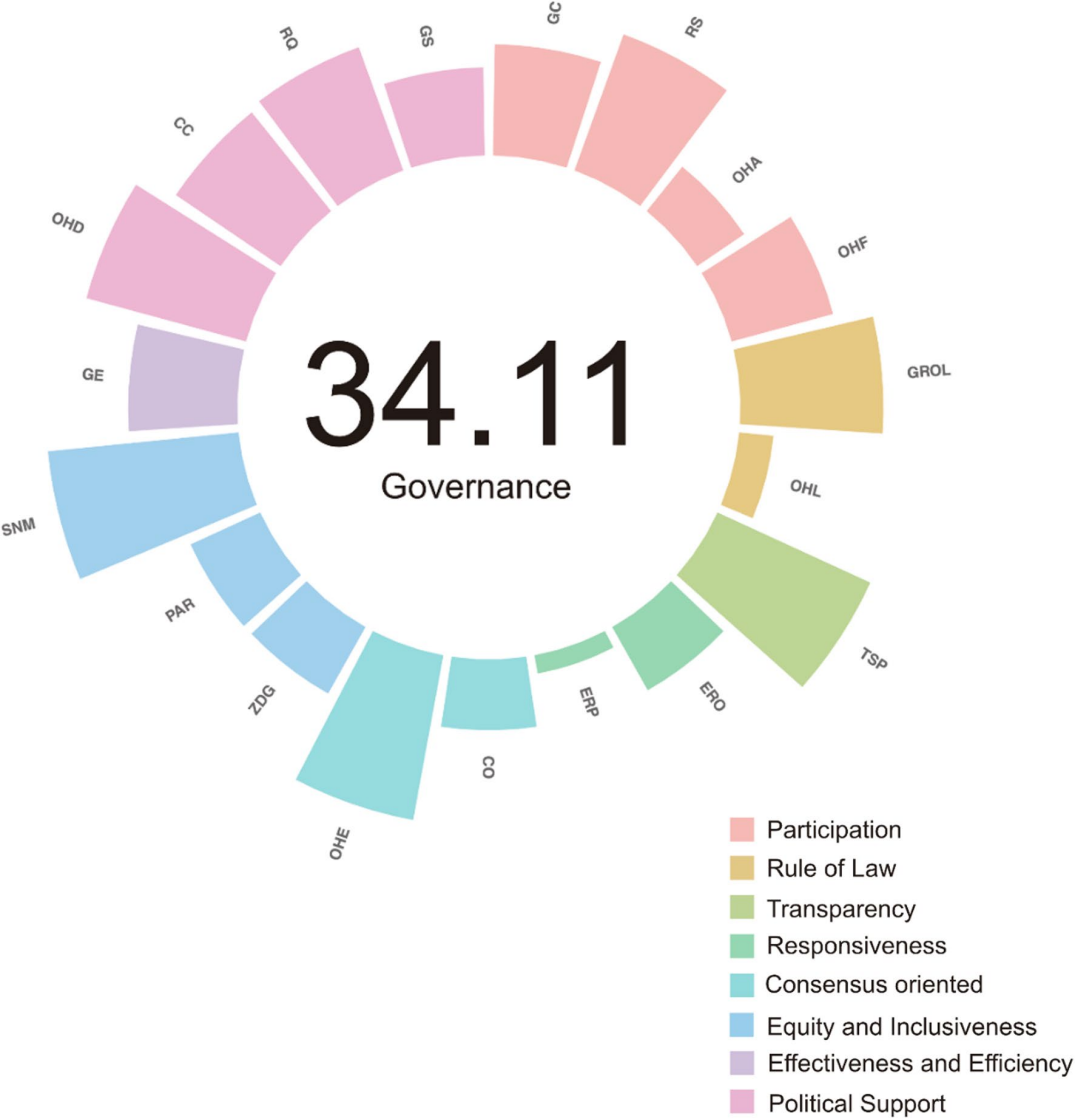
The mean score distribution of OHGI in 19 sub-indicators [*GC* global connectivity (44.10), *RC* risk communication (57.39), *OHA* One Health association (22.60), *OHF* One Health forums (41.78), *GROL* general rule of law (56.85), *OHL* One Health specialized law (13.70), *TSP* transparency (66.81), *ERO* emergency response operation (28.75), *ERP* exercising response plans (7.36), *CO* consensus oriented (28.08), *OHE* One Health education (66.44), *ZDG* zoonotic disease governance (30.28), *PAR* protected areas representativeness (30.45), *SNM* sustainable nitrogen management (76.16), *GE* government effectiveness (43.37), *OHD* One Health official department (65.75), *CC* control of corruption (50.29), *RQ* regular quality (51.99), *GS* government spending (34.92)], *OHGI* One Health governance index

Among the eight indicators, the score of rule of law was the highest (100), followed by participation (96.75), political support (92.78), effectiveness and efficiency (90.7), transparency (90.1), consensus oriented (50), responsiveness (50), and equity and inclusiveness (40.59) (Fig. 2).

While the OHGI scores for 19 sub-indicators ranged from 0 as the lowest to 100 as the highest, with the average OHGI score of 34.11 out of 100 as the highest optimal score. The OHGI scores of 19 sub-indictors with their ranges are as follows: global connectivity ranged from 23 to 87, risk communication from 0 to 100, One Health association from 0 to 100, One Health forums from 0 to 100, general rule of law from 33 to 90, One Health specialized law from 0 to 100, transparency from 21.4 to 90.1, emergency response operation from 0 to 100, exercising response plans from 0 to 87.5, consensus oriented from 0 to 100, One Health education from 0 to 100, zoonotic disease governance from 0 to 76.5, protected areas representativeness from 1.74 to 98.65, sustainable nitrogen management from 1.43 to 100, government effectiveness from 2.9 to 90.7, One Health official department from 0 to 100, control of corruption from 17.93 to 93.4, regulatory quality from 3.06 to 93.23, government spending from 2.1 to 99.5.

### OHGI score of each region and country

**Fig. 3:**
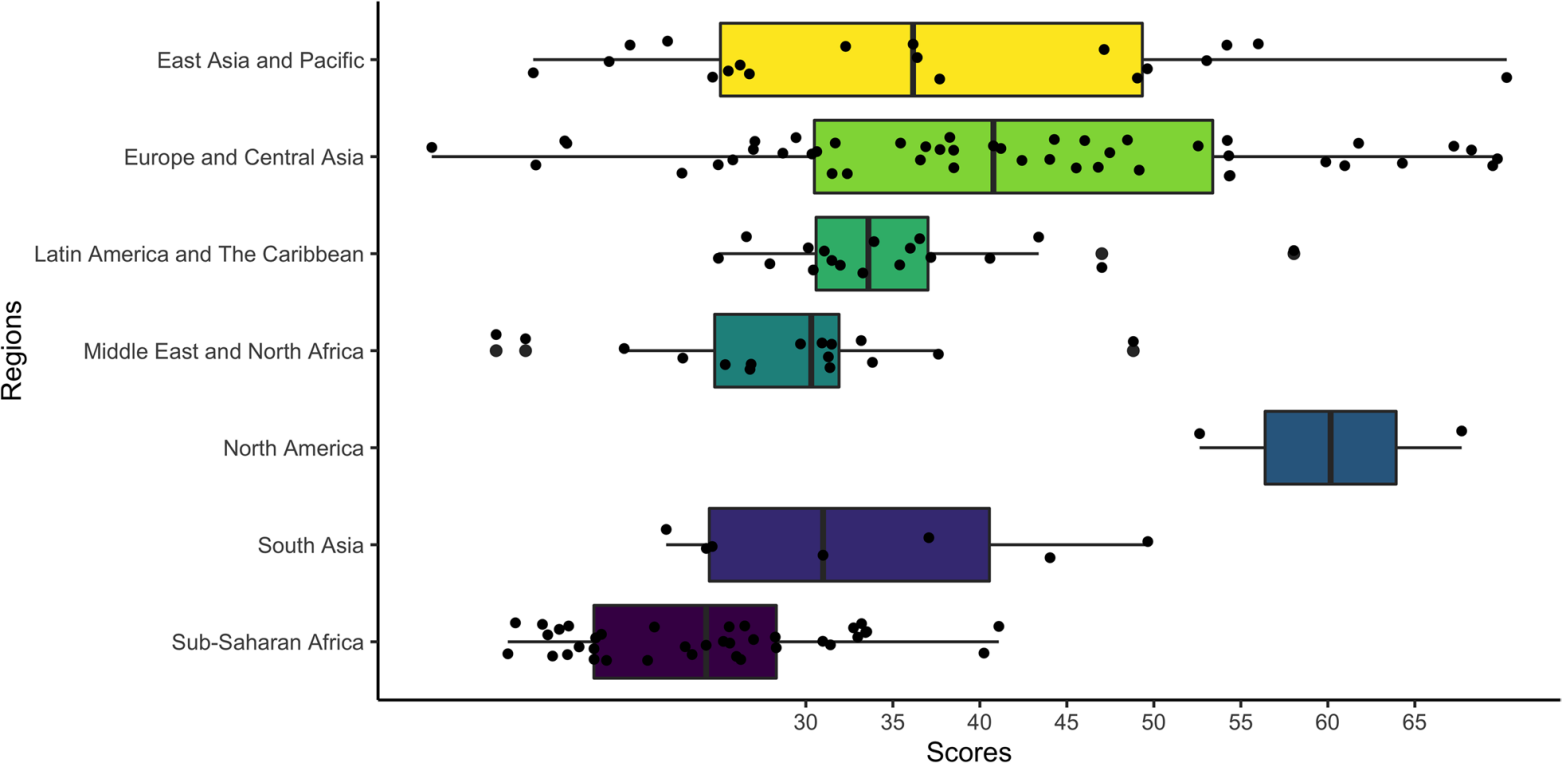
Median scores of OHGI at regional level [Northern America (60.16), Europe and Central Asia (40.78), East Asia and Pacific (36.16), Latin America and The Caribbean (33.60), South Asia (30.99), Middle East and North Africa (30.31) and Sub-Saharan Africa (24.27)], *OHGI* One Health governance index

Among the 146 countries scored in the indicator system, the median regional score for all countries was 31.49, North America (60.16) as the highest, followed by Europe and Central Asia (40.78), East Asia and Pacific (36.16), Latin America and The Caribbean (33.60), South Asia (30.99), Middle East and North Africa (30.31), and Sub-Saharan Africa (24.27) has the lowest (Fig. 3).

**Fig. 4:**
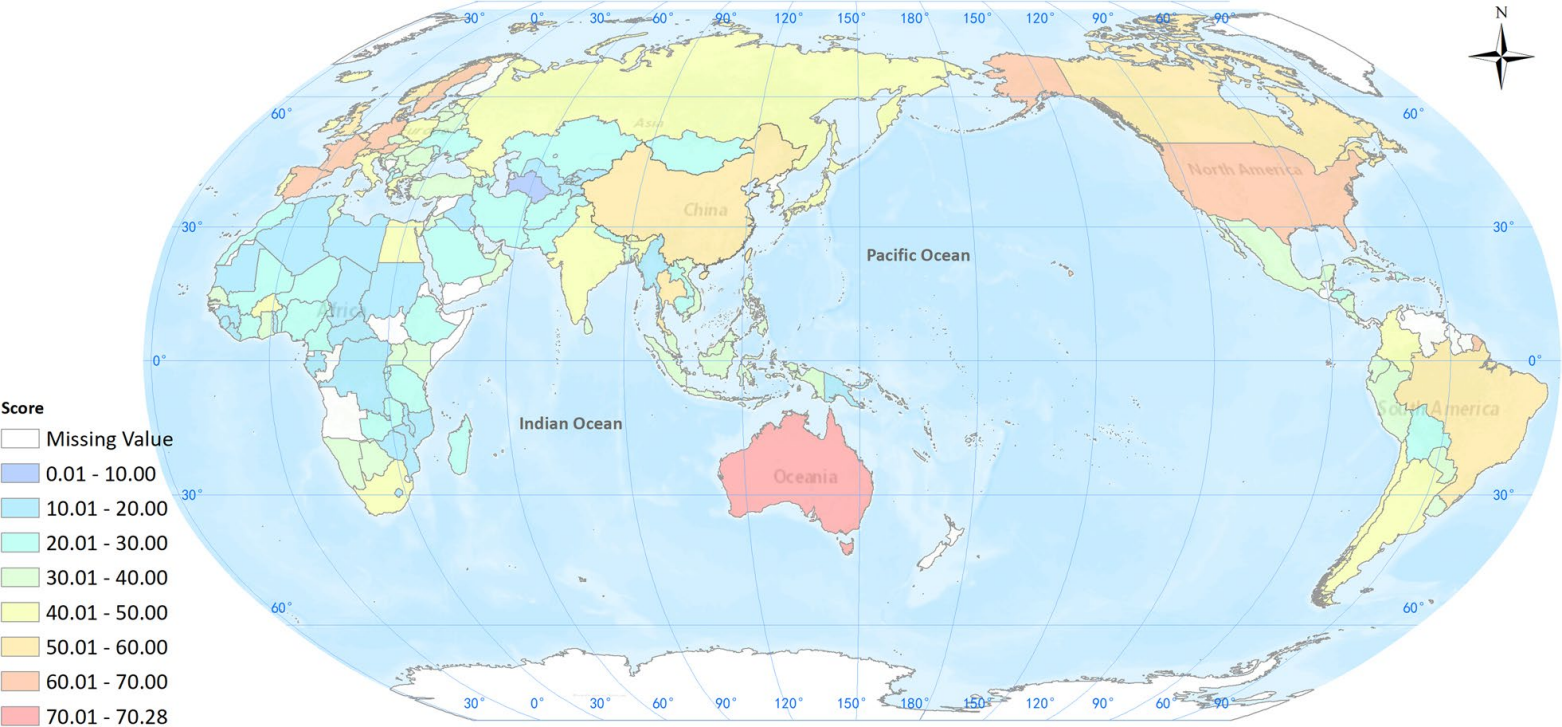
Global OHGI scores at county level [Top 20 countries with their OHGI scores are as follows: Australia (70.28), Sweden (69.75), Germany (69.48), the Netherlands (68.26), the United States of America (67.69), Finland (67.24), France (64.28), Australia (61.77), Spain (60.97), Belgium (59.88), Brazil (58.05), Thailand (56.00), United Kingdom (54.38), Norway (54.33), Switzerland (54.31), Cyprus (54.22), New Zealand (54.2), China (53.04), Canada (54.63) and Denmark (52.63)], *OHGI* One Health governance index

The OHGI scores for 146 countries of the world ranged from 8.50 as the lowest to 70.28 as the highest scores. It was showed that the top 20 countries in the list of OHGI scores are mainly from Europe, America, East Asia and the Pacific regions. While the last 30 countries are mostly from Sub-Saharan Africa (Fig. 4). The top 20 countries are Australia, Sweden, Germany, the Netherlands, the United States, Finland, France, Austria, Spain, Belgium, Brazil, Thailand, United Kingdom, Norway, Switzerland, Cyprus, New Zealand, China, Canada and Denmark, which showed that the developed countries in Europe and the United States of America are ahead of other countries in many aspects of evaluation on the One Health governance. Australia ranked as a top one had the highest score in almost all indicators and set an example in many specific measures in the field of One Health governance (Additional file 3).

The bottom 20 countries are all developing countries, of which 13 are from sub-Saharan Africa, and most of them have low scores in various indicators. This shows that these countries lack a lot of policy capacity in all links of good governance of One Health. They are almost blank in public awareness and cognition, and do not have the advanced concept of One Health. These countries are still far from the top ranked countries, and need to implement One Health good governance actions in all aspects (Additional file 4).

### Case studies on Top-3 and BRICS countries

**Fig. 5:**
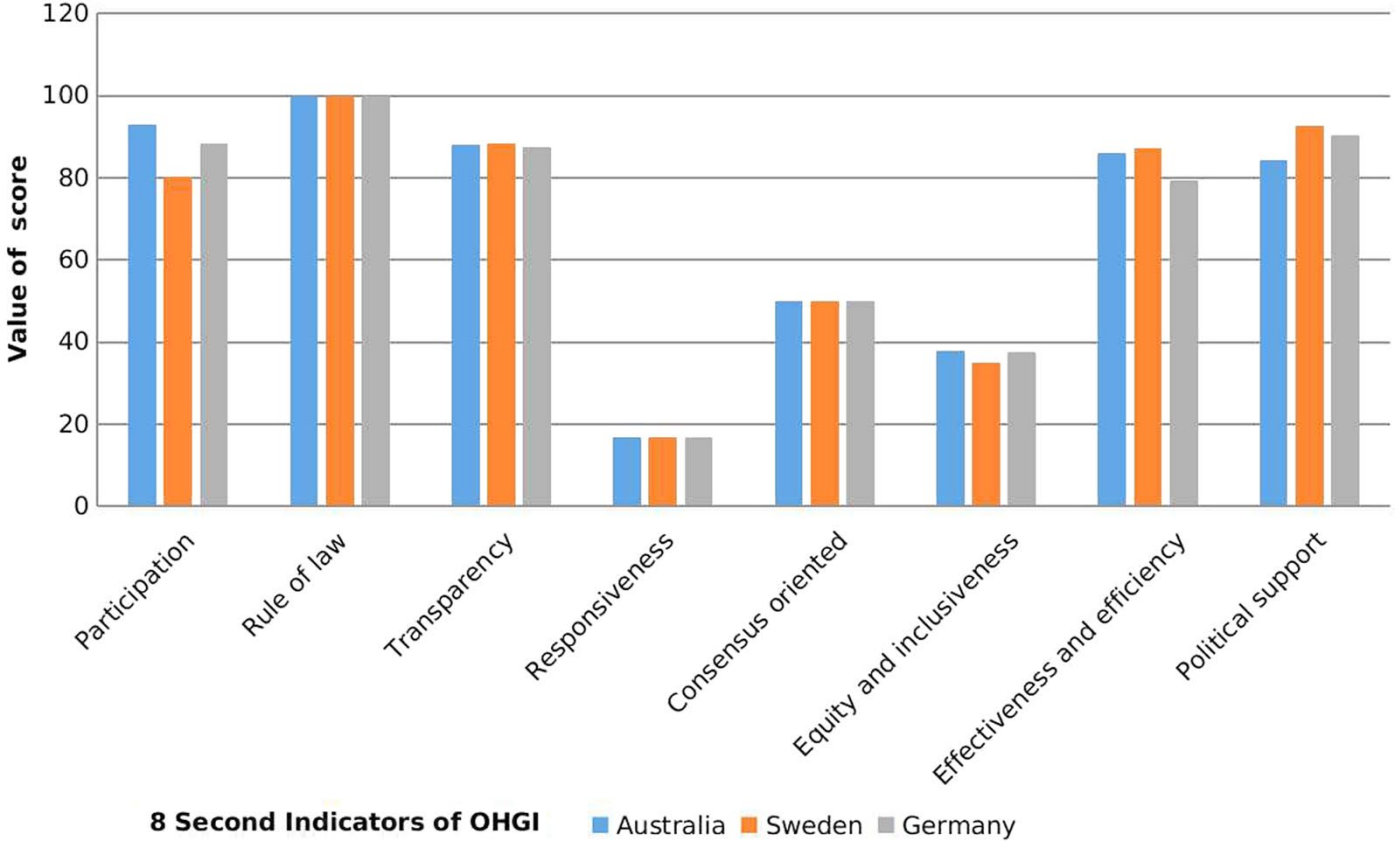
Comparison of eight indicators of the OHGI scores among top three countries including Australia, Sweden and Germany. *OHGI* One Health governance index

Among the top three countries (Australia, Sweden and Germany), it can be seen that these three countries are at the leading position in public participation, rule of law, transparency and political support. These three countries are at average level in Consensus oriented and Equity and Inclusiveness (Fig. 5). Is showed that these countries have paid more attention to the public health and have hold strong capacity in health governance as well as use laws and advanced technologies to ensure the One Health governance outcome. But in Responsiveness, these countries had relative lower scores below 20 respectively.

**Fig. 6:**
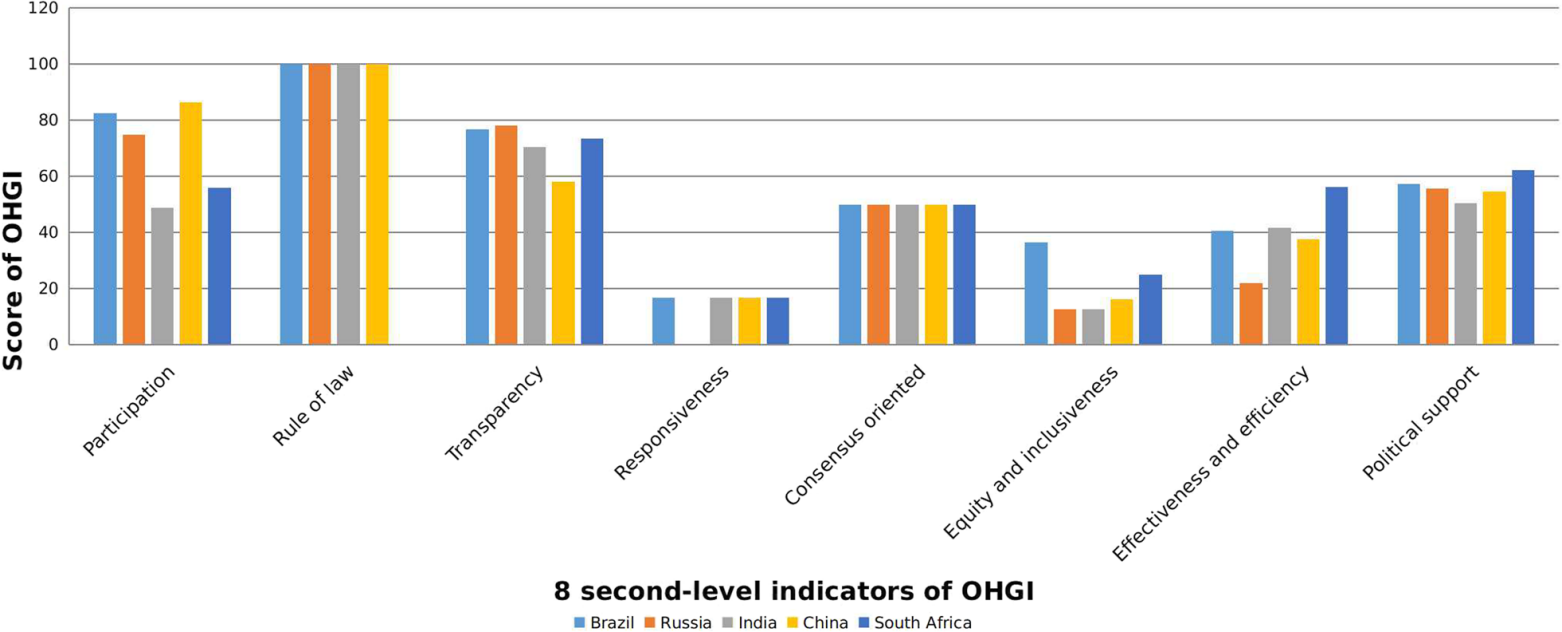
Comparison of eight indicators of OHGI scores among five BRICS countries including China, India, Russia, Brazil and South Africa. *OHGI* One Health governance index, *BRICS* Brazil, Russia, India, China, South Africa

In the case study of BRICS countries, the OHGI scores distributed in varying patterns, only three indicators including Rule of law, Responsiveness and Consensus oriented had the similar value of the OHGI scores. While the score of participation indicator was varied from 48.83 to 86.33, led by China and followed by Brazil, Russia, South Africa and India. Other indicators had uneven distribution of OHGI scores. For instance, the score of transparency was ranged from 58.2 to 78.2, led by Russia, and followed by Brazil, South Africa, China and Brazil. The score of equity & inclusiveness indicator was varied from 12.61 to 36.55, led by Brazil, and followed by South Africa, China, India and Russia. The score of effectiveness and efficiency indicator was ranged from 21.9 to 56.1, led by South Africa, and followed by India, Brazil, China and Russia. The score of political support was ranged from 50.43 to 62.36, led by South Africa, and followed by Brazil, Russia, China and India (Fig. 6).

## Discussion

### Gaps in the One Health governance

One Health governance is a leading indicator to illustrate the global health governance. In recent years, both the policy makers and the researchers at the international, regional and national levels have attached great attentions to the One Health governance, emphasizing how to promote the health in all policy by application of the good governance theory at all levels within a country [19].

Our findings showed that good governance become an important indicator to measure the One Health performance within a country or a specific region. In this assessment practice, some European countries gained OHGI scores more than 60 out of 100, indicating that these countries are relatively mature in all aspects in the implementation of One Health approach. In contrast, about 23 developing countries had scored below 20, indicating that these countries have low implementational capacity in One Health governance, mainly in the indicators of Responsiveness, Equity and Inclusiveness, etc.

In particular, the lower OHGI score in countries gained the score less than 10 indicated that its governance capacity in the field of One Health is very low and needs some technical assistance to strengthen these fields. Huge gap of OHGI scores between top (70.28) and lowest (8.5) countries was identified, which encourage us to raise more international resources to promote One Health governance capacity to close those gaps, particularly in those countries with OHGI scores less than 20, in accordance with the principle of “One World, One Health” proposed in 2004.

### Two critical fields with common concerns

The fact of huge gaps on OHGI score existed among 146 countries, showed by the study, was probably attributed to some social and economic status. For instance, almost all the countries with a higher OHGI score are from developed countries, while the lower scores are mostly presented in developing countries or underdeveloped regions. This kind of gap was also reflected mainly in the five indicators, such as public participation, rule of law, transparency, effectiveness and efficiency and policy support, which more related to the social status and policy or regulatory capacity [20, 21].

A unique feature was also observed that Responsiveness as well as Equity & Inclusiveness had relatively low OHGI scores not only in top-3 countries but also in BRICS countries, although those two groups shared different cultures and economic status. Theoretically, responsiveness refers to being able to react to the concerns of all stakeholders within a reasonable time-frame, while equity and inclusiveness refers to addressing the issues concerning justice and equity. Results from two case studies illustrated that it is not so easily to improve One Health governance unless the local governments or organizations taking One Health actions quickly, equally, and inclusively on common concerns by most residents, stakeholders, enterprises involving in the One Health program, so that most of the vulnerable and marginalized population, animals, plants and ecosystem can share the fruits of the development and have opportunities to improve and maintain holistic health at a proper way.

### Ways to fill the gap in good governance

We need to take actions to address those gaps or problems founded in this study. Firstly, for the public participation of various stakeholders in governance either through direct, legitimate intermediate institutions or representatives. It is essential to avoid any inequalities and discrimination, such as in gender, race, caste, creed, place of birth etc. So that the capacity on informed and organized participation needs to be emphasized in the One Health governance. Secondly, in terms of transparency which means all decisions must be taken and enforced in proper legal manner, the information must be freely available and directly accessible to all the people. Thirdly, Effectiveness & Efficiency means that all actions need to be taken by the optimum utilization of the resources. It is essential to sustainably use of the natural resources and the protection of the environment. Fourth, rule of law and political support indicates that the capacity to produce legislation or specific policies on One Health administrative programs should be implemented by various political actors with optimal resources.

Over all, the findings on existing gaps in the One Health governance suggested that actions need to be taken urgently. Guidance programme on One Health governance supported by the international communities could be one of more efficient ways to assistant the developing countries. Therefore, improving the capacity of One Health governance is the most important avenue to promote One Health policy and practice, particularly in developing countries.

## Conclusions

In the One Health system, good governance is an important component to evaluate a One Health system that has the function to maintain health for all human beings, animals and environment. The findings from the study indicated that the political stability, the rule of law and the economic conditions in different regions are significantly correlated with the One Health governance capacity of the region. Hence, it is urgent to take actions to improve the governance capacity on One Health, particularly in resource limited countries.

It is expected that much more One Health actions could be initiated with the assistance of this assessment tool on One Health governance. Consequently, all countries or regions can understand what they have done well and what they lack in the One Health operation measured by OHGI, a new tool developed by this study. Finally, we hope that the improvement of the One Health governance will appear there.

## Supplementary Information

**Additional file 1.** Establishment of OHGI Database.

**Additional file 2.** Protocol for calculation of One Health governance index.

**Additional file 3.** Global ranking of One Health governance index (OHGI).

**Additional file 4.** The global ranking of One Health governance index (OHGI) for each of 146 countries in the world.

## Data Availability

The full study protocol and the datasets, are available, following manuscript publication, upon request from the corresponding author (Odel Y. LI at leerhyme@outlook.com).

## Abbreviations

BRICS: Brazil, Russia, India, China, South Africa
CDC: Centers for Disease Control and Prevention
COVID-19: Coronavirus disease 2019
ECDC: European CDC
EPI: Environmental Performance Index
FAHP: Fuzzy analytical hierarchy process
FAO: Food and Agriculture Organization of the United Nations
GCI: The Global Connectivity Index
GHS: Global Health Security Index
GOHI: Global One Health Index
OHHLEP: One Health High-Level Expert Panel
OIE: World Organization for Animal Health
OHGI: One Health governance index
PARI: Protected Areas Representativeness Index
SDGs: Sustainable development goals
SNM: Sustainable Nitrogen Management Index
SPII: Statistical Performance Indicators and Index
UNEP: United Nations Environment Programme
WB: The World Bank
WGI: Worldwide governance indicator
WHO: World Health Organization
WJP: The World Justice Project
YCELP: Yale Center for Environmental Law and Policy
