# Supplementary material for: The approaching pilot for One Health governance index
Source: Infect Dis Poverty. 2023 Mar 13;12:16. doi: 10.1186/s40249-023-01067-2 (PMC10009848; doi:10.1186/s40249-023-01067-2)
Supplement: Supplementary file 1 — Additional file 1. Establishment of OHGI Database. [file 40249_2023_1067_MOESM1_ESM.docx]

**Additional 1.**

**Protocol for calculation of One Health governance index**

We have established an indicator system with 8 indicators and added 19 sub-indicators through literature review, consultancy meetings and panel discussions. We have integrated and streamlined all indicators and sorted out the logic of indicators. Finally, we established our indicator system for the One Health governance following the good governance principle (Box 1).

**Box 1. Good Governance indicator system:**

**Number of Indicators：**8 indicators, including participation, rule of law, transparency, responsiveness, consensus oriented, equity and inclusiveness, effectiveness and efficiency, and political support.

**Number of sub-indicators:** 19 sub-indicators, including global connectivity, risk communication, One Health association, One Health forums under participation; general rule of law, One Health specialized law & regulation under rule of law; transparency under transparency; emergency response operation, exercising response plans under responsiveness; consensus oriented, One Health education under participation, zoonotic disease governance, protected areas representativeness, sustainable nitrogen management under consensus oriented; government effectiveness under consensus oriented, One Health official department, control of corruption, regulatory quality, government spending under transparency. Among them, 5 sub-indicators from self-evaluated data and 14 sub-indicators from third partner database.

**Data Source：**Official data of countries from office website, official website data of international organizations, Global health security, Environmental performance index , SDGs dataset, etc., as well as self-evaluated data.

1. **1 Indicator system**

In order to solve the problem of global governance of One Health and good governance of all countries, improve the understanding of One Health governance at the national level, strengthen the government expenditure and policy investment of all countries for One Health, and increase the public participation in One Health governance, all will play a key role in One Health good governance, which involves the common governance of the government, various departments, and the public. A total of 8 indicators, including public participation, rule of law, transparency, responsiveness, consensus orientation, fairness and inclusiveness, effectiveness and efficiency, policy support were included. We have constructed the path map of good governance of One Health from the perspective of One Health (Fig 1.1). Finally, we decided to conduct a more comprehensive assessment of the One Health good governance through eight aspects.


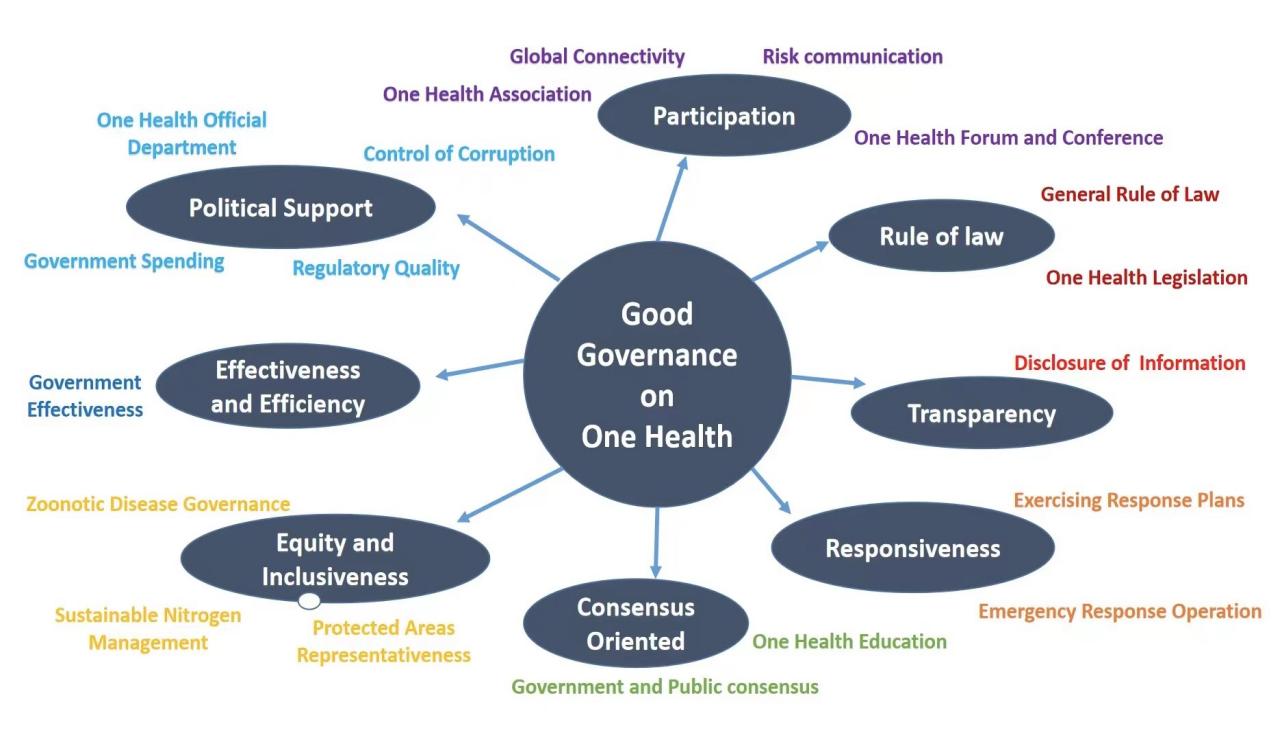


**Fig 1.** **1** Indicator system of Governance on One Health

**1.2 Sub-indicator system**

**Participation.** One health must involve all departments at all levels. It is a complex problem intersected by multiple disciplines and fields. It can not be well solved by a single department. Leading departments, departmental support, specialized agencies, citizen associations, public forums and meetings, cross departmental cooperation, cross departmental information sharing and professional training. Therefore, we set up the 4 evaluation sub-indicators to evaluate whether a country or region has the ability to link global information resources, risk communication ability, the scale of One Health industry associations and One Health public forums or high level meetings.

**Rule of law.** As we all know, in the past years, the great achievements made in the field of public health (for example, compulsory vaccination, infectious disease prevention and control, occupational protection, maternal and infant health care have made great contributions to disease prevention, health protection and health promotion) are closely related to legal intervention. It can be predicted that the health challenges we face in the 21st century (for example, the establishment of a reasonable medical and health care system, the prevention and control of emerging infectious diseases, the response to chronic non communicable diseases, tobacco control, environmental protection, life technology regulation, the elimination of health gap, etc.) will not make substantive progress without laws. For the intervention of various factors affecting One Health. The law can be implemented in two modes: direct regulation (for example, authorizing relevant departments to take health intervention measures to endow citizens with health rights and obligations) and indirect regulation (for example, restraining the development of tobacco and other industries harmful to health through taxation, punishing public nuisance enterprises such as environmental pollution through tort litigation, etc.).

**Transparency.** The transparency of governance refers to aim at the openness and availability of data for One Health management. For this indicator, the research group adopts the SDG data source.

**Responsiveness.** The response mechanism plays a key role in the One Health management in public health emergencies. Emergency response operation and emergency plan drill are the investigation fields of public health emergency mechanism, and these two main factors are evaluated. We evaluateEmergency response operation and Exercising response plans. For this indicator, the research group make a reference to the Global health security report.

**Consensus orientation.** Consensus orientation consists of the consensus mechanism of the government and the public on public health related fields and two sub-indicators of general consensus orientation and One health Education.

**Equity and inclusiveness.** This indicator specifically evaluated by the data of zoonosis treatment, regional environmental protection treatment and sustainable nitrogen management. The effectiveness and efficiency of One Health governance are the assessment factors of good governance.

**Effectiveness and efficiency.** The effectiveness and efficiency of One Health governance are the assessment factors of good governance. Therefore, we have set six three-level indicators, including human resources, material resources, financial resources, information and technology resources, facility distribution, voice and accountability, to evaluate the effectiveness and efficiency of global governance.

**Political support.** The political support of all countries is the reason that must be considered when investigating the One Health governance. The political support of all countries is the reason that must be considered when examining the One Health governance. The research team divided political support into four three-level indicators: special government institutions for One Health, corruption management, policy quality and government expenditure. The data sources are mainly official data such as WB, who and SDG reports.

It should be noted that several self-evaluated indicator data on One health, such as One health association, One health forum, specialized legislation on One health, One health education and One Health official institutions (the original data are from the national reports of the World Health Organization, the official websites of disease control departments of various countries and other databases). These data are obtained by the research group in the form of national official data and professional and authoritative legal databases, It is also an important innovation of this paper.

**1.3 Weighting for each sub-indicators**

From the perspective of One Health research, we cited the food and Agriculture Organization of the United Nations, the World Health Organization, the global health security index, the SDG data report, as well as the European CDC (ECDC), the World Health Organization, the global justice project, Johns Hopkins University public health data, Yale University environmental data, Huawei data and the data published on the official websites of various countries, and set up a total of 27 indicatiors, including 8 indicators and 19 sub-indicators. We hope to build a part of the One Health index in a more comprehensive dimension, evaluate the advantages and disadvantages of each country and region by scoring the relevant scores of One Health governance, and provide reasonable suggestions(Table 1).

A fuzzy analytical hierarchy process (FAHP) was adopted to assign the weights for most of the indicators. We used a fuzzy comparison matrix based on the judgements of different experts to generate the weight matrix of indicators. We conducted two rounds of investigations among our expert advisory committee and collected the experts opinions by several rounds of  interrogation on the comparison of relative importance between indicators. Therefore, we obtained the weights of the eight indicators in the form of questionnaire survey. The weight of sub indicators is distributed equally.

**Table 1.2** OHGI Index system

| **OHgi** | **First indicator weight** | **indicator** | **Second indicator weight** | **Sub-indicator** | **Sub-indicator weight** | **Source** |
| --- | --- | --- | --- | --- | --- | --- |
| Good Governance | **1** | Participation | 10.97% | Global Connectivity | 25.00% | From third partner database |
|  |  |  |  | Risk communication | 25.00% | From third partner database |
|  |  |  |  | One Health Association | 25.00% | From self-evaluated data |
|  |  |  |  | One Health Forums | 25.00% | From self-evaluated data |
|  |  | Rule of law | 15.75% | General rule of law | 50.00% | From third partner database |
|  |  |  |  | One Health Specilized law & Regulation | 50.00% | From self-evaluated data |
|  |  | Transparency | 9.98% | Transparency | 100.00% | From third partner database |
|  |  | Responsiveness | 12.56% | Emergency response operation | 50.00% | From third partner database |
|  |  |  |  | Exercising response plans | 50.00% | From third partner database |
|  |  | Consensus oriented | 10.84% | General consensus oriented | 50.00% | From third partner database |
|  |  |  |  | One Health Education | 50.00% | From self-evaluated data |
|  |  | Equity and inclusiveness | 13.79% | Zoonotic disease governance | 33.33% | From third partner database |
|  |  |  |  | Protected Areas Representativeness | 33.33% | From third partner database |
|  |  |  |  | Sustainable Nitrogen Management | 33.33% | From third partner database |
|  |  | Effectiveness and Efficiency | 13.18% | Government effectiveness | 100.00% | From third partner database |
|  |  | Political support | 12.93% | One Health Official Department | 25.00% | From self-evaluated data |
|  |  |  |  | Control of Corruption | 25.00% | From third partner database |
|  |  |  |  | Regulatory Quality | 25.00% | From third partner database |
|  |  |  |  | Government spending | 25.00% | From third partner database |

**1.4 Limitations**

The World Health Organization, WB, SDG data, European CDC, World Health Organization, national CDC and other multi-party databases are international well-known organizations or large country databases. Therefore, the data of developed countries are relatively complete, while some data of developing countries are missing. Secondly, there are only data of nearly 1-2 years for many indicators, and there is a lack of continuous and dynamic change results in the evaluation. In the future, we will continue to optimize various indicators and increase the collection of missing data to make version of OHGI updated. On the other hand, there is still a lack of data from a country on the One Health global governance and local governance. Because the One Health includes the important connotation of global health, this part of data collection is quite difficult. Even if the data are comparable, it is worth discussing. Therefore, the scientificity and universality of the indicators will be optimized in the future.
