# Supplementary material for: The approaching pilot for One Health governance index
Source: Infect Dis Poverty. 2023 Mar 13;12:16. doi: 10.1186/s40249-023-01067-2 (PMC10009848; doi:10.1186/s40249-023-01067-2)
Supplement: Supplementary file 2 — Additional file 2. Protocol for calculation of One Health governance index. [file 40249_2023_1067_MOESM2_ESM.docx]

**Additional 2. Establishment of OHGI Database**

**2.1 OHGI primary database**

**Extracted database.** 14 indicators from all evaluation indicators are obtained through existing official data. These data sources are: Huawei global connectivity index(GCI), Johns Hopkins University global health security index(GHS Index), Yale University environmental performance index(EPI), SDG report, World bank official data, etc.

**Self-evaluated database.** The members of the research group screened and reviewed the official data of 146 countries, including official websites of government departments, national official education departments, national health departments, national universities, classified databases and legal databases for the five indicators of One health organization, One health forum, special legislation on One health, One health education and One health government departments as self-evaluated data sources. These are important research innovation of this study.

**2.2 Data sources**

Through 8 indicators, 19 sub-indicators with bottom data, OHGI use multiple dimensions to comprehensively assess the degree of good governance on One Health. OHGI Data Sources came from authoritative institutions websites and scientific reports, such as the Food and Agriculture Organization, the World Health Organization, as well as the European CDC (ECDC), the Global Connectivity Index (GCI), the SDG report, the World Justice Project (WJP), Global Health Security Index (GHS), Statistical Performance Indicators and Index, Protected Areas Representativeness Index (PARI), Sustainable Nitrogen Management Index (SNM) , and data published on national official websites have integrated and established the framework of the global governance index for One Health.

**Table 2.1** OHGI Indicator sources

| **Second indicator** | **Sub-indicator** | **Source** | **Institutions of Source Database** |
| --- | --- | --- | --- |
| Participation | Global Connectivity | the Global Connectivity Index (GCI) | HUAWEI |
|  | Risk communication | Global Health Security Index (GHS 3.5) | Johns Hopkins University |
|  | One Health Association | Official Websites | Official Websites |
|  | One Health Forums | Official Websites | Official Websites |
| Rule of law | General rule of law | the World Justice Project (WJP) | American Bar Association |
|  | One Health Specialized law & Regulation | Authoritative Legal Databases | HeinOnLine, etc. |
| Transparency | Transparency | Statistical Performance Indicators and Index (SPII) | the World Bank |
| Responsiveness | Emergency response operation | Global Health Security Index (GHS 3.3) | Johns Hopkins University |
|  | Exercising response plans | Global Health Security Index (GHS 3.2) | Johns Hopkins University |
| Consensus oriented | Linking Authorities | Global Health Security Index (GHS 3.4) | Johns Hopkins University |
|  | One Health Education | Official Websites | Official Websites |
| Equity and inclusiveness | Zoonotic disease governance | Global Health Security Index (GHS 1.2) | Johns Hopkins University |
|  | Protected Areas Representativeness | Protected Areas Representativeness Index (PARI) | Yale Center for Environmental Law and Policy |
|  | Sustainable Nitrogen Management | Sustainable Nitrogen Management Index (SNM) | Yale Center for Environmental Law and Policy |
| Effectiveness and Efficiency | Government effectiveness | Worldwide governance indicator (WGI-GE) | the World Bank |
| Political support | One Health Official Department | Official Websites | Official Websites |
|  | Control of Corruption | Worldwide governance indicator (WGI-CC) | the World Bank |
|  | Regulatory Quality | Worldwide governance indicator (WGI-RQ) | the World Bank |
|  | Government spending | Government spending on health & education  ( SDG report Note Indicator17) | United Nation |

**2.3 OHGI score database**

**Indicator of Participation** express the information connectivity and risk communication capabilities of countries around the world. Huawei data is used for the evaluation of global connectivity, Johns Hopkins University global health security index(GHS Index) is used for the evaluation of risk communication, and the evaluation of One health Association and One health forum are evaluated by self-evaluated data sources;

**Indicator of Rule of law** express the comprehensive legal capacity of countries all over the world. The general rule of law indicator adopts the global justice project, and the special legislation on one health indicator adopts its own indicators;

**Indicator of Transparency** refers to the openness and availability of data for total health management. For this indicator, the research group adopts the SDG data source；

**Indicator of Responsiveness** plays a key role in the whole health management in public health emergencies. Emergency response operation and emergency plan drill are the investigation fields of public health emergency mechanism, and these two main factors are evaluated. For this indicator, the research group adopts the GHS Index；

**Indicator of Consensus oriented** consists of the consensus mechanism of the government and the public on public health related fields and two four level indicators of special education for one health. For this indicator, the research group adopts the GHS Index and self-evaluated data;

**Indicator of Effectiveness and efficiency** of total health governance are the assessment factors of good governance, which are derived from the public health data of Johns Hopkins University;

**Indicator of Equity and inclusiveness** comes from the environmental performance index of Yale University, which makes a specific evaluation on the treatment of zoonosis, regional environmental protection and sustainable nitrogen management. For this indicator, the research group adopts Yale University environmental performance index(EPI);

**Indicator of** **Political support** is an important factor in the evaluation of national health governance. The research team divided political support into four three-level indicators: special government institutions for One health, corruption management, policy quality and government expenditure, etc. For this indicator, the research group adopts the World bank data and self-evaluated data.

**Figure 2.2**  The distribution of data sources with institutions

The distribution of data sources was investigated. Among them, the data of Participation are extracted from Huawei data, Johns Hopkins University public health data and self-evaluated data sources; The data of the rule of law adopts the global justice project and global professional legal database: HeinOnLine, LexisNexis and Westlaw; The original data of Transparency comes from the SDG report; Responsiveness and Effectiveness & Efficiency data are from Johns Hopkins University; Data of Consensus oriented are from Johns Hopkins University public health data; The data of Equity and inclusiveness are extracted from Yale University environmental performance index; The data of political support comes from World Bank official website, SDG report and self-evaluated index evaluation.
