# Supplementary material for: The approaching pilot for One Health governance index
Source: Infect Dis Poverty. 2023 Mar 13;12:16. doi: 10.1186/s40249-023-01067-2 (PMC10009848; doi:10.1186/s40249-023-01067-2)
Supplement: Supplementary file 3 — Additional file 3. Global ranking of One Health governance index (OHGI). [file 40249_2023_1067_MOESM3_ESM.docx]

**Additional 3. OHGI Rankings**

3. 1 The mean score distribution of OHGI in 19 indicators


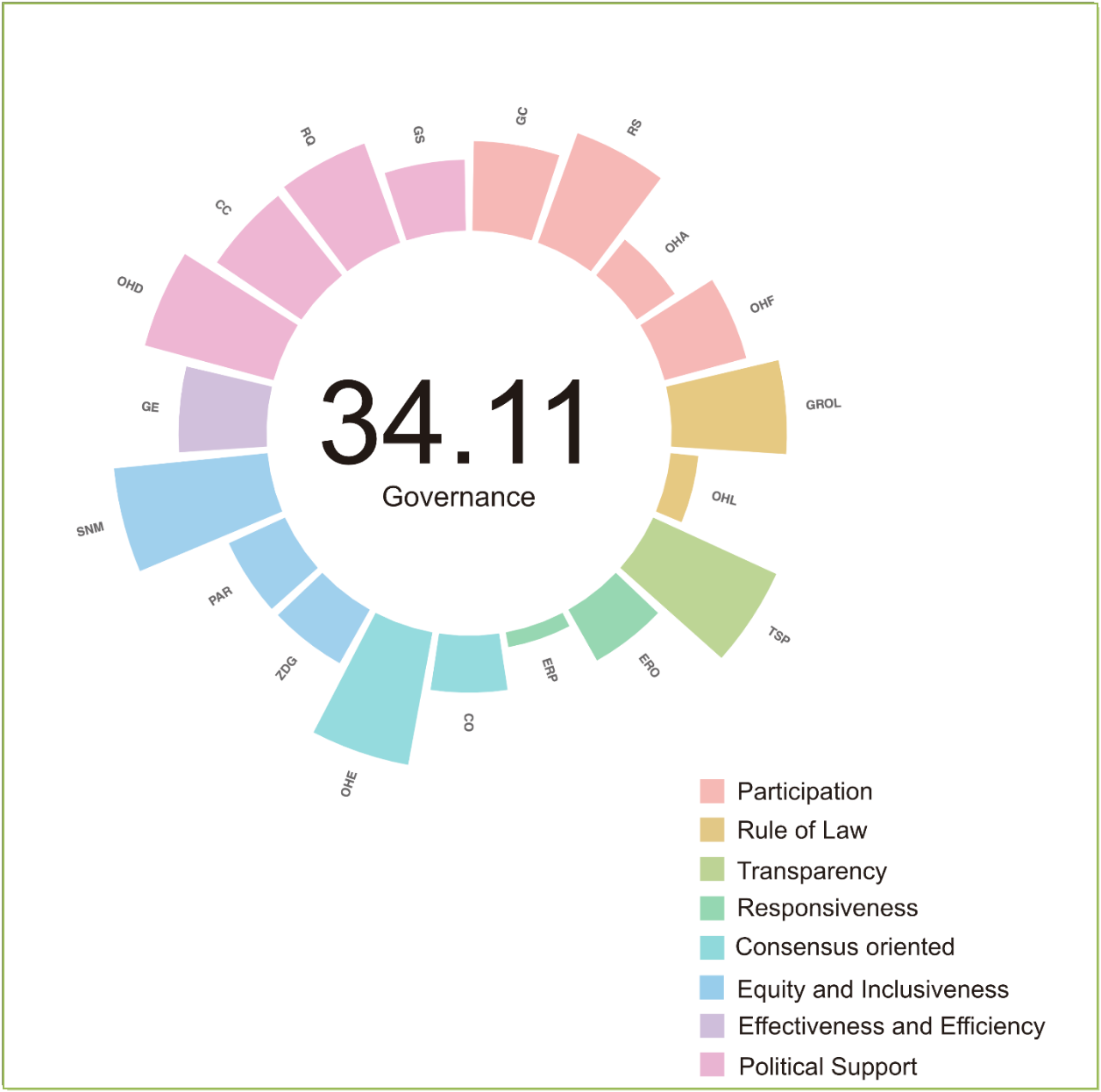


**Fig. 3** The mean score distribution of OHGI in 19 indicators

3.2 Global score Map of One Health Governance inedex

Among the 146 countries scored in our index system, the country with the first score is Australia, with a score of 70.28. The average score for all countries was 34.11. The countries with higher overall regional scores are those in Europe and Central Asia, East Asia and the Pacific and North America. Sub Saharan Africa has the lowest overall regional score.


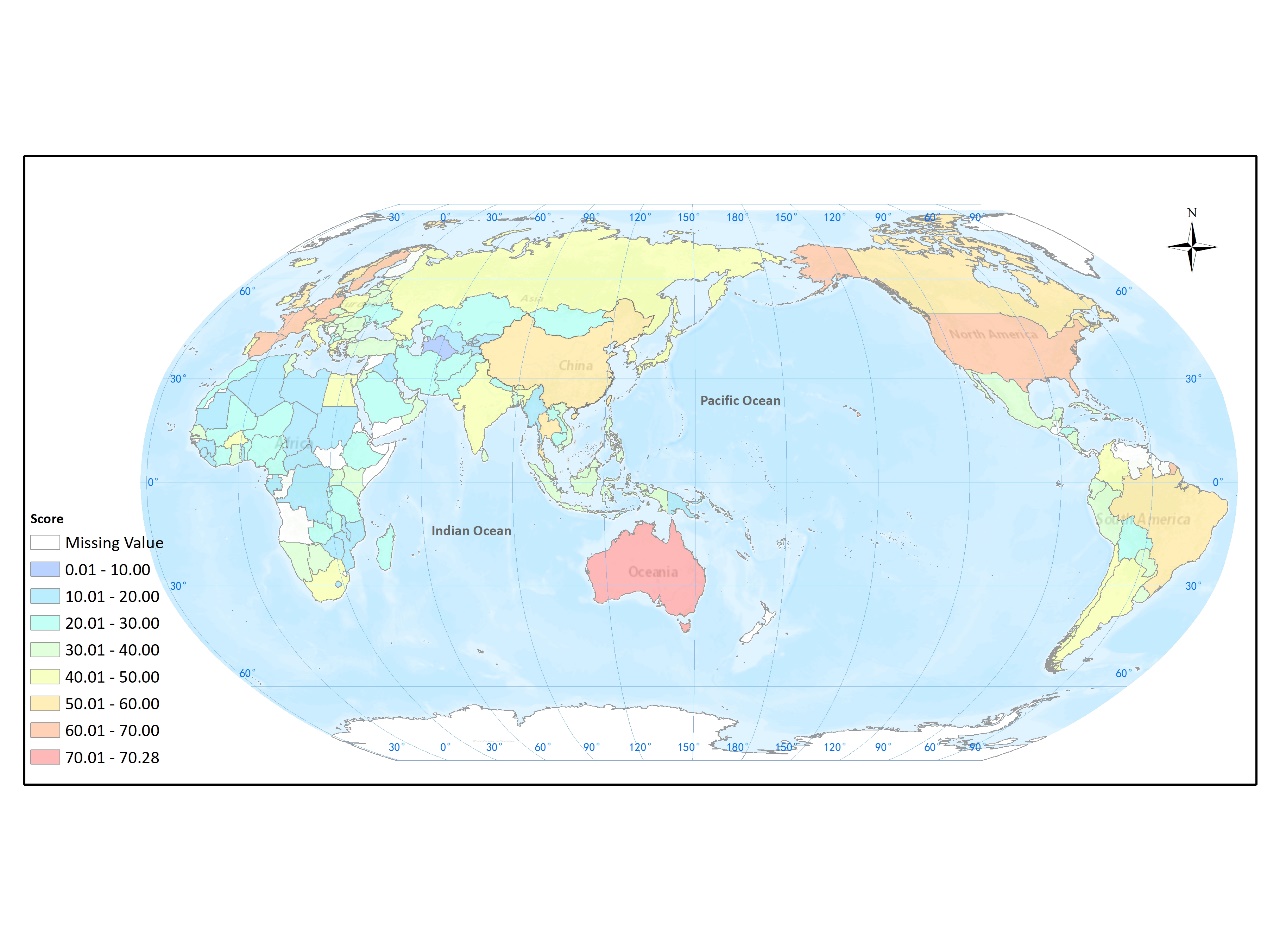


Fig. 4 Global score Map of One Health Governance index
