# Supplementary material for: The approaching pilot for One Health governance index
Source: Infect Dis Poverty. 2023 Mar 13;12:16. doi: 10.1186/s40249-023-01067-2 (PMC10009848; doi:10.1186/s40249-023-01067-2)
Supplement: Supplementary file 4 — Additional file 4. The global ranking of One Health governance index (OHGI) for each of 146 countries in the world. [file 40249_2023_1067_MOESM4_ESM.docx]

**Additional 4. The global ranking of One Health governance index (OHGI) for each of 146 countries in the world**

4.1 Score of each countries

Most of the countries ranking 1-30 are from East Asia and the Pacific, Europe and Central Asia and North America. On the list, only Brazil is in Latin America. It can be seen that the overall performance of countries in these three regions is better than that in other regions.

**Table 4.1** Global ranking of of One Health governance index (OHGI) for each of 143 countries of the world

| **Global Rank** | | | | | | | | |
| --- | --- | --- | --- | --- | --- | --- | --- | --- |
| **Rank** | **Country** | **Score** | **Rank** | **Country** | **Score** | **Rank** | **Country** | **Score** |
| 1 | Australia | 70.28 | 51 | Turkey | 36.89 | 101 | Mongolia | 26.22 |
| 2 | Sweden | 69.75 | 52 | Romania | 36.58 | 102 | Rwanda | 26.01 |
| 3 | Germany | 69.48 | 53 | Peru | 36.54 | 103 | Albania | 25.8 |
| 4 | Netherlands | 68.26 | 54 | Indonesia | 36.4 | 104 | Zambia | 25.63 |
| 5 | United States of America | 67.69 | 55 | Philippines | 36.16 | 105 | Tanzania | 25.59 |
| 6 | Finland | 67.24 | 56 | Barbados | 36 | 106 | Cambodia | 25.54 |
| 7 | France | 64.28 | 57 | Armenia | 35.44 | 107 | Morocco | 25.37 |
| 8 | Austria | 61.77 | 58 | Uruguay | 35.39 | 108 | Niger | 25.26 |
| 9 | Spain | 60.97 | 59 | Cuba | 33.92 | 109 | Honduras | 24.97 |
| 10 | Belgium | 59.88 | 60 | Malta | 33.83 | 110 | Azerbaijan | 24.96 |
| 11 | Brazil | 58.05 | 61 | Ghana | 33.5 | 111 | Fiji | 24.64 |
| 12 | Thailand | 56 | 62 | Botswana | 33.43 | 112 | Pakistan | 24.62 |
| 13 | United Kingdom | 54.38 | 63 | Ecuador | 33.27 | 113 | Afghanistan | 24.28 |
| 14 | Norway | 54.33 | 64 | Senegal | 33.2 | 114 | Ethiopia | 24.27 |
| 15 | Switzerland | 54.31 | 65 | Qatar | 33.18 | 115 | Madagascar | 23.46 |
| 16 | Cyprus | 54.22 | 66 | Mauritius | 32.98 | 116 | Cabo Verde | 23.06 |
| 17 | New Zealand | 54.2 | 67 | Uganda | 32.74 | 117 | Bahrain | 22.93 |
| 18 | China | 53.04 | 68 | Czech Republic | 32.38 | 118 | Kazakhstan | 22.88 |
| 19 | Canada | 52.63 | 69 | Viet Nam | 32.29 | 119 | Laos | 22.05 |
| 20 | Denmark | 52.55 | 70 | Paraguay | 31.98 | 120 | Nepal | 21.97 |
| 21 | India | 49.65 | 71 | Lithuania | 31.69 | 121 | Burundi | 21.3 |
| 22 | Japan | 49.63 | 72 | Serbia | 31.51 | 122 | Mali | 20.9 |
| 23 | Russia | 49.16 | 73 | Costa Rica | 31.49 | 123 | Papua New Guinea | 19.89 |
| 24 | Singapore | 49.05 | 74 | Oman | 31.48 | 124 | Algeria | 19.56 |
| 25 | Egypt | 48.82 | 75 | Kenya | 31.41 | 125 | Myanmar | 18.69 |
| 26 | Iceland | 48.48 | 76 | United Arab Emirates | 31.38 | 126 | Mozambique | 18.54 |
| 27 | Ireland | 47.47 | 77 | Tunisia | 31.29 | 127 | Lesotho | 18.25 |
| 28 | South Korea | 47.14 | 78 | Belize | 31.06 | 128 | Togo | 17.92 |
| 29 | Chile | 47.01 | 79 | Sri Lanka | 30.99 | 129 | Sudan | 17.83 |
| 30 | Slovakia | 46.8 | 80 | Namibia | 30.97 | 130 | Zimbabwe | 17.82 |
| 31 | Poland | 46.03 | 81 | Jordan | 30.93 | 131 | Sierra Leone | 16.97 |
| 32 | Portugal | 45.54 | 82 | Bulgaria | 30.63 | 132 | Dem. Rep. Congo | 16.37 |
| 33 | Estonia | 44.28 | 83 | Trinidad and Tobago | 30.43 | 133 | Central African Republic | 16.3 |
| 34 | Bhutan | 44.03 | 84 | Belarus | 30.35 | 134 | Uzbekistan | 16.26 |
| 35 | Luxembourg | 44.02 | 85 | Nicaragua | 30.14 | 135 | Kyrgyzstan | 16.14 |
| 36 | Colombia | 43.38 | 86 | Iran | 29.69 | 136 | Liberia | 15.83 |
| 37 | Italy | 42.42 | 87 | Ukraine | 29.43 | 137 | Guinea | 15.44 |
| 38 | Greece | 41.21 | 88 | Moldova | 28.68 | 138 | Malawi | 15.17 |
| 39 | Burkina Faso | 41.09 | 89 | Cameroon | 28.3 | 139 | Mauritania | 14.86 |
| 40 | Georgia | 40.78 | 90 | Nigeria | 28.24 | 140 | Tajikistan | 14.48 |
| 41 | Argentina | 40.58 | 91 | Bolivia | 27.93 | 141 | Timor-Leste | 14.33 |
| 42 | South Africa | 40.24 | 92 | North Macedonia | 27.07 | 142 | Iraq | 13.89 |
| 43 | Croatia | 38.5 | 93 | Benin | 26.99 | 143 | Gabon | 13.31 |
| 44 | Latvia | 38.49 | 94 | Montenegro | 26.98 | 144 | Chad | 12.87 |
| 45 | Hungary | 38.27 | 95 | Saudi Arabia | 26.85 | 145 | Libya | 12.2 |
| 46 | Slovenia | 37.71 | 96 | Lebanon | 26.79 | 146 | Turkmenistan | 8.5 |
| 47 | Malaysia | 37.69 | 97 | Brunei Darussalam | 26.75 |  |  |  |
| 48 | Israel | 37.62 | 98 | Dominican Republic | 26.58 |  |  |  |
| 49 | Mexico | 37.18 | 99 | Cote d'Ivoire | 26.49 |  |  |  |
| 50 | Bangladesh | 37.07 | 100 | Seychelles | 26.26 |  |  |  |

4.2 Major Leaders

The top 20 countries are all developing countries, of which 13 are from Europe and Central Asia, and most of them have high scores in various indicators. This shows that they have a lot of policy capacity in good governance of One Health. They have the ability to cooperate with One Health governance departments, and they are good at legislating on One Health issue because of their advanced concept of One Health. These countries have already implemented some One Health good governance actions in some aspects.

**Table 4.2** Top regional ranking (20th)

| **Ranking** | **Country** | **Region** | **Good Governance** | **Participation** | **Rule of law** | **Transparency** | **Responsiveness** | **Consensus oriented** | **Equity & inclusiveness** | **Effectiveness & efficiency** | **Political Support** |
| --- | --- | --- | --- | --- | --- | --- | --- | --- | --- | --- | --- |
| 1 | Australia | East Asia and Pacific | 70.28 | 93 | 100 | 88.2 | 16.65 | 50 | 37.97 | 86 | 84.45 |
| 2 | Sweden | Europe and Central Asia | 69.75 | 80.42 | 100 | 88.5 | 16.65 | 50 | 35 | 87.1 | 92.78 |
| 3 | Germany | Europe and Central Asia | 69.48 | 88.33 | 100 | 87.5 | 16.65 | 50 | 37.66 | 79.3 | 90.29 |
| 4 | Netherlands | Europe and Central Asia | 68.26 | 89.58 | 100 | 88 | 16.65 | 50 | 28.14 | 83.1 | 85.5 |
| 5 | United States of America | North America | 67.69 | 96.75 | 100 | 79.1 | 33.35 | 50 | 29.82 | 70.9 | 76.87 |
| 6 | Finland | Europe and Central Asia | 67.24 | 64.83 | 100 | 88.5 | 16.65 | 50 | 33.93 | 87.1 | 87.77 |
| 7 | France | Europe and Central Asia | 64.28 | 56.05 | 100 | 86.3 | 16.65 | 50 | 33.2 | 78.1 | 84.36 |
| 8 | Austria | Europe and Central Asia | 61.77 | 58.17 | 100 | 89.1 | 16.65 | 50 | 38.19 | 75.1 | 58.9 |
| 9 | Spain | Europe and Central Asia | 60.97 | 61.08 | 100 | 88.9 | 16.65 | 50 | 25.5 | 65.4 | 74.29 |
| 10 | Belgium | Europe and Central Asia | 59.88 | 54 | 100 | 82.4 | 16.65 | 50 | 32.88 | 75 | 58.77 |
| 11 | Brazil | Latin America and The Caribbean | 58.05 | 82.58 | 100 | 76.8 | 16.65 | 50 | 36.55 | 40.7 | 57.34 |
| 12 | Thailand | East Asia and Pacific | 56 | 61.5 | 100 | 76.1 | 33.35 | 50 | 28.62 | 37.3 | 55.77 |
| 13 | United Kingdom | Europe and Central Asia | 54.38 | 87.5 | 0 | 83.2 | 33.35 | 50 | 34.31 | 79.3 | 86.65 |
| 14 | Norway | Europe and Central Asia | 54.33 | 84.92 | 0 | 90.1 | 16.65 | 50 | 30.98 | 90.6 | 90.81 |
| 15 | Switzerland | Europe and Central Asia | 54.31 | 91.07 | 0 | 87 | 16.65 | 50 | 40.59 | 90.7 | 77.48 |
| 16 | Cyprus | Europe and Central Asia | 54.22 | 26.42 | 100 | 80.8 | 16.65 | 50 | 21.7 | 62 | 65.41 |
| 17 | New Zealand | East Asia and Pacific | 54.2 | 88.83 | 0 | 85.3 | 16.65 | 50 | 36.77 | 87.4 | 87.48 |
| 18 | China | East Asia and Pacific | 53.04 | 86.33 | 100 | 58.2 | 16.65 | 50 | 16.17 | 37.7 | 54.71 |
| 19 | Canada | North America | 52.63 | 88.33 | 0 | 87.5 | 16.65 | 50 | 21.69 | 90.1 | 87.24 |
| 20 | Denmark | Europe and Central Asia | 52.55 | 90.08 | 0 | 86.1 | 16.65 | 50 | 28.29 | 80.4 | 89.49 |

4.3 Major laggards

The bottom 20 countries are all developing countries, of which 13 are from sub Saharan Africa, and most of them have low scores in various indicators. This shows that they lack a lot of policy capacity in all links of good governance of One Health. They neither have the ability to cooperate with One Health governance departments, nor can they reach the level of legislative technology. They are almost blank in public awareness and cognition, and do not have the advanced concept of One Health. These countries are still far from the top ranked countries, and need to implement One Health good governance actions in all aspects.

**Table 4.3** Ranking of lower regions (20th)

| **Rank** | **Country** | **Region** | **Good Governance** | **Participation** | **Rule of law** | **Transparency** | **Responsiveness** | **Consensus oriented** | **Equity & inclusiveness** | **Effectiveness & efficiency** | **Political Support** |
| --- | --- | --- | --- | --- | --- | --- | --- | --- | --- | --- | --- |
| 127 | Lesotho | Sub-Saharan Africa | 18.25 | 20.08 | 0 | 55.2 | 0 | 0 | 5 | 38 | 35.66 |
| 128 | Togo | Sub-Saharan Africa | 17.92 | 12.57 | 0 | 60.9 | 16.65 | 0 | 19.39 | 22.1 | 20.48 |
| 129 | Sudan | Sub-Saharan Africa | 17.83 | 22.7 | 0 | 39.2 | 16.65 | 50 | 3.8 | 13 | 12.37 |
| 130 | Zimbabwe | Sub-Saharan Africa | 17.82 | 16.31 | 0 | 61.7 | 16.65 | 0 | 27.43 | 10.3 | 19.96 |
| 131 | Sierra Leone | Sub-Saharan Africa | 16.97 | 27.07 | 0 | 52.8 | 16.65 | 0 | 5.03 | 22.1 | 22.43 |
| 132 | Dem. Rep. Congo | Sub-Saharan Africa | 16.37 | 16.69 | 0 | 33.8 | 0 | 50 | 13.25 | 10 | 19.59 |
| 133 | Central African Republic | Sub-Saharan Africa | 16.3 | 18.77 | 0 | 52.3 | 0 | 0 | 18.22 | 10.9 | 38.72 |
| 134 | Uzbekistan | Europe and Central Asia | 16.26 | 24.07 | 0 | 54.9 | 16.65 | 0 | 4.15 | 21.1 | 19.82 |
| 135 | Kyrgyzstan | Europe and Central Asia | 16.14 | 23.55 | 0 | 57.83 | 0 | 0 | 10.82 | 22 | 25.19 |
| 136 | Liberia | Sub-Saharan Africa | 15.83 | 22.98 | 0 | 49.4 | 0 | 0 | 19.62 | 22.4 | 19.97 |
| 137 | Guinea | Sub-Saharan Africa | 15.44 | 19.13 | 0 | 45.9 | 16.65 | 0 | 12.97 | 18.3 | 18.25 |
| 138 | Malawi | Sub-Saharan Africa | 15.17 | 12.52 | 0 | 56.5 | 0 | 0 | 13.95 | 22.4 | 24.34 |
| 139 | Mauritania | Sub-Saharan Africa | 14.86 | 13.82 | 0 | 48.1 | 16.65 | 0 | 5.42 | 21.7 | 21 |
| 140 | Tajikistan | Europe and Central Asia | 14.48 | 12.37 | 0 | 55.8 | 16.65 | 0 | 15.43 | 7.1 | 18.24 |
| 141 | Timor-Leste | East Asia and Pacific | 14.33 | 15.95 | 0 | 51 | 0 | 0 | 10.88 | 19.3 | 25.71 |
| 142 | Iraq | Middle East and North Africa | 13.89 | 14.15 | 0 | 34.8 | 0 | 0 | 7.68 | 16.9 | 42.36 |
| 143 | Gabon | Sub-Saharan Africa | 13.31 | 15.52 | 0 | 28.1 | 0 | 0 | 12.48 | 33 | 19.56 |
| 144 | Chad | Sub-Saharan Africa | 12.87 | 15.51 | 0 | 38.5 | 0 | 0 | 6.57 | 9.9 | 39.05 |
| 145 | Libya | Middle East and North Africa | 12.2 | 10.67 | 0 | 21.4 | 0 | 50 | 1.71 | 9.6 | 14.82 |
| 146 | Turkmenistan | Europe and Central Asia | 8.5 | 16.83 | 0 | 23.5 | 16.65 | 0 | 3.18 | 2.9 | 10.66 |

4.4 Score of each countries at a regional level

**Table 4.4.1** Ranking of Europe and Central Asia

| **Europe and Central Asia** | | |
| --- | --- | --- |
| Rank | Country | Score |
| 2 | Sweden | 69.75 |
| 3 | Germany | 69.48 |
| 4 | Netherlands | 68.26 |
| 6 | Finland | 67.24 |
| 7 | France | 64.28 |
| 8 | Austria | 61.77 |
| 9 | Spain | 60.97 |
| 10 | Belgium | 59.88 |
| 13 | United Kingdom | 54.38 |
| 14 | Norway | 54.33 |
| 15 | Switzerland | 54.31 |
| 16 | Cyprus | 54.22 |
| 20 | Denmark | 52.55 |
| 23 | Russia | 49.16 |
| 26 | Iceland | 48.48 |
| 27 | Ireland | 47.47 |
| 30 | Slovakia | 46.8 |
| 31 | Poland | 46.03 |
| 32 | Portugal | 45.54 |
| 33 | Estonia | 44.28 |
| 35 | Luxembourg | 44.02 |
| 37 | Italy | 42.42 |
| 38 | Greece | 41.21 |
| 40 | Georgia | 40.78 |
| 43 | Croatia | 38.5 |
| 44 | Latvia | 38.49 |
| 45 | Hungary | 38.27 |
| 46 | Slovenia | 37.71 |
| 51 | Turkey | 36.89 |
| 52 | Romania | 36.58 |
| 57 | Armenia | 35.44 |
| 68 | Czech Republic | 32.38 |
| 71 | Lithuania | 31.69 |
| 72 | Serbia | 31.51 |
| 82 | Bulgaria | 30.63 |
| 84 | Belarus | 30.35 |
| 87 | Ukraine | 29.43 |
| 88 | Moldova | 28.68 |
| 92 | North Macedonia | 27.07 |
| 94 | Montenegro | 26.98 |
| 103 | Albania | 25.8 |
| 110 | Azerbaijan | 24.96 |
| 118 | Kazakhstan | 22.88 |
| 134 | Uzbekistan | 16.26 |
| 135 | Kyrgyzstan | 16.14 |
| 140 | Tajikistan | 14.48 |
| 146 | Turkmenistan | 8.5 |

**Table 4.4.2** Middle East and North Africa ranking

| **Middle East and North Africa** | | |
| --- | --- | --- |
| Rank | Country | Score |
| 6 | Egypt | 48.82 |
| 45 | Israel | 37.62 |
| 46 | Malta | 33.83 |
| 48 | Qatar | 33.18 |
| 55 | Oman | 31.48 |
| 60 | United Arab Emirates | 31.38 |
| 64 | Tunisia | 31.29 |
| 71 | Jordan | 30.93 |
| 73 | Iran | 29.69 |
| 80 | Saudi Arabia | 26.85 |
| 83 | Lebanon | 26.79 |
| 102 | Morocco | 25.37 |
| 108 | Bahrain | 22.93 |
| 129 | Algeria | 19.56 |
| 130 | Iraq | 13.89 |
| 137 | Libya | 12.2 |

**Table 4.4.3** Ranking of Latin America and the Caribbean

| **Latin America and The Caribbean** | | |
| --- | --- | --- |
| Rank | Country | Score |
| 11 | Brazil | 58.05 |
| 29 | Chile | 47.01 |
| 36 | Colombia | 43.38 |
| 41 | Argentina | 40.58 |
| 49 | Mexico | 37.18 |
| 53 | Peru | 36.54 |
| 56 | Barbados | 36 |
| 58 | Uruguay | 35.39 |
| 59 | Cuba | 33.92 |
| 63 | Ecuador | 33.27 |
| 70 | Paraguay | 31.98 |
| 73 | Costa Rica | 31.49 |
| 78 | Belize | 31.06 |
| 83 | Trinidad and Tobago | 30.43 |
| 85 | Nicaragua | 30.14 |
| 91 | Bolivia | 27.93 |
| 98 | Dominican Republic | 26.58 |
| 109 | Honduras | 24.97 |

**Table 4.4.4** North America ranking

| **North America** | | |
| --- | --- | --- |
| Rank | Country | Score |
| 5 | United States of America | 67.69 |
| 19 | Canada | 52.63 |

**Table 4.4.5** Ranking of East Asia and the Pacific

| **East Asia and Pacific** | | |
| --- | --- | --- |
| Rank | Country | Score |
| 1 | Australia | 70.28 |
| 12 | Thailand | 56 |
| 17 | New Zealand | 54.2 |
| 18 | China | 53.04 |
| 22 | Japan | 49.63 |
| 25 | Singapore | 49.05 |
| 29 | South Korea | 47.14 |
| 47 | Malaysia | 37.69 |
| 54 | Indonesia | 36.4 |
| 55 | Philippines | 36.16 |
| 69 | Viet Nam | 32.29 |
| 97 | Brunei Darussalam | 26.75 |
| 101 | Mongolia | 26.22 |
| 106 | Cambodia | 25.54 |
| 111 | Fiji | 24.64 |
| 119 | Laos | 22.05 |
| 123 | Papua New Guinea | 19.89 |
| 125 | Myanmar | 18.69 |
| 141 | Timor-Leste | 14.33 |

**Table 4.4.6** Ranking of South Asia

| **South Asia** | | |
| --- | --- | --- |
| Rank | Country | Score |
| 21 | India | 49.65 |
| 34 | Bhutan | 44.03 |
| 50 | Bangladesh | 37.07 |
| 79 | Sri Lanka | 30.99 |
| 112 | Pakistan | 24.62 |
| 113 | Afghanistan | 24.28 |
| 120 | Nepal | 21.97 |

**Table 4.4.7** Sub Saharan Africa ranking

| **Sub-Saharan Africa** | | |
| --- | --- | --- |
| Rank | Country | Score |
| 39 | Burkina Faso | 41.09 |
| 42 | South Africa | 40.24 |
| 61 | Ghana | 33.5 |
| 62 | Botswana | 33.43 |
| 64 | Senegal | 33.2 |
| 66 | Mauritius | 32.98 |
| 67 | Uganda | 32.74 |
| 75 | Kenya | 31.41 |
| 80 | Namibia | 30.97 |
| 89 | Cameroon | 28.3 |
| 90 | Nigeria | 28.24 |
| 93 | Benin | 26.99 |
| 99 | Cote d'Ivoire | 26.49 |
| 100 | Seychelles | 26.26 |
| 102 | Rwanda | 26.01 |
| 104 | Zambia | 25.63 |
| 105 | Tanzania | 25.59 |
| 108 | Niger | 25.26 |
| 114 | Ethiopia | 24.27 |
| 115 | Madagascar | 23.46 |
| 116 | Cabo Verde | 23.06 |
| 121 | Burundi | 21.3 |
| 122 | Mali | 20.9 |
| 126 | Mozambique | 18.54 |
| 127 | Lesotho | 18.25 |
| 128 | Togo | 17.92 |
| 129 | Sudan | 17.83 |
| 130 | Zimbabwe | 17.82 |
| 131 | Sierra Leone | 16.97 |
| 132 | Dem. Rep. Congo | 16.37 |
| 133 | Central African Republic | 16.3 |
| 136 | Liberia | 15.83 |
| 137 | Guinea | 15.44 |
| 138 | Malawi | 15.17 |
| 139 | Mauritania | 14.86 |
| 143 | Gabon | 13.31 |
| 144 | Chad | 12.87 |
